# Hepatitis B virus inhibits the in vivo and in vitro synthesis and secretion of apolipoprotein C3

**DOI:** 10.1186/s12944-017-0607-2

**Published:** 2017-11-13

**Authors:** Chengliang Zhu, Hengcheng Zhu, Hui Song, Limin Xu, Longxuan Li, Fang Liu, Xinghui Liu

**Affiliations:** 1 Department of Clinical Laboratory, Renmin Hospital of Wuhan University, Wuhan, Hubei 430060 People’s Republic of China; 2 Department of Clinical Laboratory, Shanghai Gongli Hospital, the Second Military Medical University, Pudong New Area, Shanghai, 200135 China; 3 Department of Neurology, Shanghai Gongli Hospital, the Second Military Medical University, Pudong New Area, Shanghai, 200135 China; 4 The State Key Laboratory of Virology, College of Life Sciences, Wuhan University, Wuhan, Hubei 430072 People’s Republic of China

**Keywords:** Hepatitis B virus, Apolipoprotein C3, Triglyceride, Very-low-density lipoprotein

## Abstract

**Background:**

Hepatitis B virus (HBV) infection in the body can damage liver cells and cause disorders in blood lipid metabolism. Apolipoprotein C3 (ApoC3) plays an important role in the regulation of lipid metabolism, but no study on the HBV regulation of ApoC3 has been reported. This purpose of this study was to investigate the effect of HBV on ApoC3 expression and its regulatory mechanism.

**Methods:**

The expression levels of ApoC3 mRNA and protein in the human hepatoma cell lines HepG2 and HepG2.2.15 were determined using real-time quantitative reverse transcription polymerase chain reaction (RT-qPCR) and Western blot. The HepG2 cells were co-transfected with the ApoC3 gene promoter and either HBV-infected clone pHBV1.3 or its individual genes. The changes in luciferase activity were assayed. The expression levels of ApoC3 mRNA and protein were determined using RT-qPCR and Western blot. The content of ApoC3 in the supernatant of the cultured cells was determined using an enzyme-linked immunosorbent assay (ELISA). The sera were collected from 149 patients with HBV infection and 102 healthy subjects at physical examination as the normal controls. The serological levels of ApoC3 in the HBV group and the normal control group were determined using ELISA. The contents of serum triglyceride (TG) and very-low-density lipoprotein (VLDL) in the HBV patients and the normal control were determined using an automatic biochemical analyser.

**Results:**

The expression levels of ApoC3 mRNA and protein were lower in the HepG2.2.15 cells than in the HepG2 cells. pHBV1.3 and its X gene could inhibit the activity of the ApoC3 promoter and its mRNA and protein expression. The serum levels of ApoC3, VLDL and TG were 65.39 ± 7.48 μg/ml, 1.24 ± 0.49 mmol/L, and 0.46 ± 0.10 mmol/L in the HBV patients and 41.02 ± 6.88 μg/ml, 0.76 ± 0.21 mmol/L, 0.29 ± 0.05 mmol/L in the normal controls, respectively, statistical analysis revealed significantly lower serum levels of ApoC3, VLDL and TG in HBV patients than in the normal controls (*P* < 0.05).

**Conclusion:**

HBV can inhibit the in vivo and in vitro synthesis and secretion of ApoC3.

## Background

Hepatitis B virus (HBV) is a hepatotropic DNA virus. After HBV infects the body, approximately 10% of the infection can progress to a chronic infection. Currently, approximately 250 million people in the world are suffering from chronic hepatitis B or are chronic HBV carriers, and chronic HBV infection is a major factor leading to liver fibrosis and liver cancer. Approximately 3 million people with chronic HBV infection die of chronic B hepatitis, cirrhosis or liver cancer every year [1, 2].

The apolipoprotein (Apo) family is large and mainly includes apolipoproteins A, B, C, and E, which each are a special type of protein. Lipid must be combined with Apo in the process of normal transport [3]. Of the C family members, apolipoprotein C3 (ApoC3) is seen at the highest concentration; its gene is located on the long arm q23 region of chromosome 11 and includes 4 exons and 3 introns. ApoC3 forms a gene family with Apo A1 and A4 and is mostly synthesized in the liver, with a small amount synthesized in the small intestine [4]. As a main component of triacylglycerol-rich lipoprotein (TRL) and high-density lipoprotein (HDL), ApoC3 plays an important role in the regulation of lipid metabolism and can regulate the decomposition and metabolism of TRL in hypertriglyceridemia [5–7].

It was reported that HBV down-regulated the expression of ApoA1, ApoA5, ApoB and up-regulated the expression of ApoM [8–11].The main purpose of this study is to investigate the effect of HBV on the regulation of ApoC3 expression and its mechanism by in vivo and in vitro experiments.

## Materials and methods

### Study subjects

A total of 149 patients with clinical diagnosis of hepatitis B infection in the outpatient and inpatient departments were recruited, including 105 males and 44 females, with a mean age of 44.7 ± 12.5 years. Meanwhile, 102 healthy subjects who received a physical examination were recruited as the control group, which included 70 males and 32 females, with a mean age of 43.1 ± 11.5 years. Hepatotropic virus infections hepatitis A virus (HAV), hepatitis C virus (HCV) and hepatitis D virus (HDV), as well as other diseases that can cause metabolic abnormalities, were excluded in all patients. All subjects signed the informed consent, and this study was approved by the Ethics Committee of the hospital.

### Cell culture and transfection

HepG2 and HepG2.2.15 cells were cultured in RPMI-1640 medium containing 10% foetal bovine serum, 100 U/mL penicillin and 100 mg/L streptomycin in a 37 °C incubator at 5% CO_2_. Before the transfection, HepG2 cells were inoculated into 24-well plates or 6-well plates. When the cell confluence reached approximately 80%, plasmid DNA and Lipofectin2000 transfection reagent were diluted in serum-free and penicillin-streptomycin-free RPMI-1640 medium at room temperature for 20 min. The prepared transfection solution was added to the HepG2 cell culture medium, and the cells were incubated in the CO_2_ incubator.

### Determination of luciferase activity

After transfection, the HepG2 cells were cultured for 48 h, the supernatant was discarded, and the cells were washed with phosphate-buffered saline (PBS) before they were lysed with cell lysate buffer. After the cells were completely lysed, 20 μL of cell lysate and 100 μL of luciferase substrate were mixed and measured with a Luminometer. Each experiment was repeated three times.

### RT-qPCR

HepG2 and HepG2.2.15 cells were harvested, and 1 mL of TRIzol® reagent was added to extract the total RNA. After being treated with DNase, 1 μg of RNA was used to synthesize cDNA by reverse transcriptase M-MLV. The ApoC3 gene was quantitatively measured using a SYBR® Green™ qPCR mix kit. The primers for ApoC3 were as follows: (sense) - 5’GTT ACA TGA AGC ACG CCA CC3’ and (antisense) - 5’CAC GGC TGA AGT TGG TCT GA3’. GAPDH was used as the internal reference [12].

### Western blotting

HepG2 and HepG2.2.15 cells were ultrasonically disrupted, and the supernatant was obtained. After an equal volume of loading buffer was added, the sample was boiled for 5 min and subjected to 12% sodium dodecyl sulfate–polyacrylamide gel electrophoresis (SDS-PAGE). After electrophoresis, the separated proteins were transferred onto a nitrocellulose (NC) membrane. After blocking with skim milk, 1:2000 polyclonal antibody for ApoC3 and 1:5000 goat anti-rabbit secondary antibody were each added, followed by the appropriate amount of electrochemiluminescence (ECL) solution. The membrane was placed in a gel imaging system to visualize the protein.

### ELISA detection

The levels of ApoC3 in the serum and cell supernatant were measured by an enzyme-linked immunosorbent assay (ELISA) according to the manual of the ApoC3 ELISA kit. Each experiment was repeated three times.

### Biochemical testing

The serum triglyceride (TG) level was measured according to the enzymatic principle using an automated biochemical analyser, and the very-low-density lipoprotein (VLDL) level was calculated according to Friedwald’s equation.

### Statistical analysis

SPSS20.0 statistical software was used for data processing. All data are presented as X̅ ± S. The comparison between groups was performed using t-tests. Difference with *P* < 0.05 were considered statistically significant.

## Results

### Baseline characteristics

The clinical baseline characteristics of the participants are listed in Table 1. The serum HDL-C, LDL-C, TC, ApoA1 and ApoB were significantly lower in HBV patients comparing with healthy individuals (*p* < 0.05), while there was no significant difference in gender, age and BMI between the two groups (*P* > 0.05).

**Table 1:** Baseline characteristics of the subjects enrolled in the present study

Clinical parameters	Healthy individuals (*n* = 102)	HBV patients (*n* = 149)	*P*
Age (years)	43.1 ± 11.5	44.7 ± 12.5	0.437
Gender (male/female)	70/32	105/44	0.397
BMI	24.7 ± 1.4	25.3 ± 1.6	0.485
ALT(IU/l)	<30	215.2 ± 262.5	<0.01
AST(IU/l)	<30	139.5 ± 182.4	<0.01
TC (mmol/L)	4.28 ± 0.58	3.86 ± 0.75	<0.01
HDL-C(mmol/L)	1.62 ± 0.27	1.38 ± 0.32	<0.01
LDL-C (mmol/L)	2.63 ± 0.56	2.25 ± 0.55	<0.01
ApoA1(g/mL)	1.38 ± 0.16	0.93 ± 0.27	<0.01
ApoB(g/mL)	0.88 ± 0.24	0.73 ± 0.18	<0.01

### HBV inhibits the expression levels of ApoC3 mRNA and protein

**Fig. 1:**
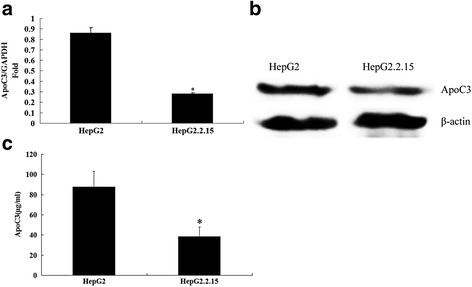
Effect of HBV on ApoC3 mRNA and protein expression levels in HepG2 cells. **a** The total RNA of HepG2 and HepG2.2.15 cells was extracted, and the ApoC3 mRNA level was quantitatively determined by RT-qPCR; **b** The expression levels of ApoC3 protein in HepG2 and HepG2.2.15 were determined using Western blot; **c** The contents of ApoC3 in the supernatants of HepG2 and HepG2.2.15 cells were determined by ELISA. *P < 0.005

Using RT-qPCR and Western blot, the expression levels of ApoC3 mRNA and protein in HepG2 and HepG2.2.15 cells were determined. The results showed that the ApoC3 expression in HepG2.2.15 cells was lower than that in HepG2 control cells (Fig. 1a and b). Furthermore, the contents of ApoC3 in the HepG2 and HepG2.2.15 cell supernatants were determined by ELISA. The results showed that the expression levels of ApoC3 in HepG2 and HepG2.2.15 cells were 46.15 ± 9.58 μg/ml and 28.02 ± 8.46 *μ*g/ml, respectively, with a statistically significant difference (*P* < 0.05, Fig. 1c), indicating that HBV can inhibit the expression of ApoC3 in HepG2 cells.

### HBV inhibits the gene promoter activity and mRNA/protein expression of ApoC3

**Fig. 2:**
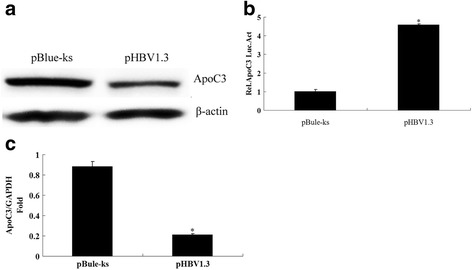
Effect of pHBV1.3 on the promoter activity and mRNA and protein expression levels of ApoC3. **a** At 48 h after 0.6 μg of pHBV1.3 and its control plasmid pBlue-ks were each co-transfected with 0.2 μg of ApoC3 gene promoter pApoC3-Luc into HepG2 cells, the changes in luciferase activity were determined using a luminometer; each experiment was repeated three times; **b** at 48 h after 4 μg of pHBV1.3 and its control plasmid pBlue-ks were each transfected into HepG2 cells, the effect of HBV on the expression of ApoC3 mRNA was determined by RT-qPCR; **c** The expression levels of ApoC3 protein in HepG2 and HepG2.2.15 cells were determined by Western blot; **d** The content of ApoC3 in the supernatant of HepG2 cells was determined by ELISA. *P < 0.005

pHBV1.3, an HBV-infected clone, and the ApoC3 gene promoter pApoC3-Luc were co-transfected into HepG2 cells, with pBlue-ks as the control plasmid. After 48 h, the activity of the luciferase reporter gene was determined. The results showed that the activity of luciferase was 189.42 ± 27.3 μg/protein after transfection with pHBV1.3 and 857.36 ± 55.6 *μ*g/protein after transfection with pBlue-ks; the difference was statistically significant (Fig. 4, *P* < 0.05), indicating that pHBV1.3 was able to inhibit ApoC3 gene promoter activity, as shown in Fig. 2a.

Furthermore, pHBV1.3 and its control plasmid pBlue-ks were transfected into HepG2 cells. After 48 h, the expression levels of ApoC3 mRNA and protein were determined by RT-qPCR and Western blot. The results showed that the expression levels of ApoC3 mRNA and protein were reduced in the HepG2 cells transfected with pHBV1.3 compared with the transfection control (Fig. 2b and c). Using ELISA, the content of ApoC3 in the cell supernatant was determined. The content of ApoC3 in the supernatant of HepG2 cells transfected with pHBV1.3 was 36.84 ± 11.26 μg/ml, which was lower than 95.53 ± 17.31 *μ*g/ml in the supernatant of HepG2 cells transfected with pBlue-ks (Fig. 2d).

### HBV inhibits ApoC3 expression through its X gene

**Fig. 3:**
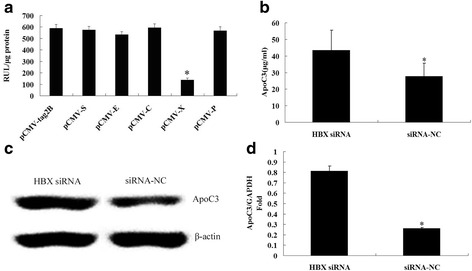
HBV inhibited ApoC3 expression through its X gene. **a** HepG2 cells were transfected with pCMV-S, pCMV-E, pCMV-C, pCMV-X, pCMV-P and pCMV-tag2B, the changes in luciferase activity were determined using a luminometer at 48 h post-transfection, each experiment was repeated three times. **b** HepG2.2.15 cells were transfected with the HBX siRNA or the negative control siRNA(siRNA-NC) for 24 h. ApoC3 mRNA level was quantitatively determined by RT-qPCR; **c** The expression levels of ApoC3 protein in HepG2.2.15 cells were determined by Western blot; **d** The content of ApoC3 in the supernatant of HepG2.2.15 cells was determined by ELISA. *P < 0.005

HepG2 cells were co-transfected with all of the individual plasmid eukaryotic expression vectors containing the HBV genome and the ApoC3 gene promoter pApoC3-Luc, respectively, pCMV-tag2B was set as the control [11]. The results showed that the HBX significantly down-regulated the promoter activity of ApoC3 gene (Fig. 3a).

Furthermore, HepG2.2.15 cells were transfected with HBX siRNA or its control (siRNA-NC) [13]. Results showed that ApoC3 mRNA and protein expression were significantly increased after transfection with HBX siRNA (Fig. 3b and c), and the content of ApoC3 in the supernatant of HepG2.2.15 cells transfected with HBX siRNA was 43.26 ± 12.27 μg/ml, which was higher than 27.65 ± 7.88 μg/ml in the supernatant of HepG2.2.15 cells transfected with siRNA-NC (Fig. 3d), indicating that HBX inhibits the expression of ApoC3.

### Serum ApoC3 level of HBV patients was decreased

**Fig. 4:**
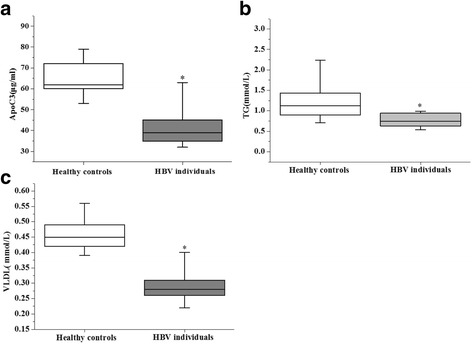
Measurement of ApoC3, TG and VLDL in HBV patients and normal controls. **a** Comparison of the serum levels of ApoC3 in HBV patients and normal controls; **b** Comparison of the serum levels of TG in HBV patients and normal controls; **c** Comparison of the serum levels of VLDL in HBV patients and normal controls. *P < 0.005

The serum levels of ApoC3, TG and VLDL in the HBV patient group and the normal control group were tested. The results showed that the serum levels of ApoC3, TG and VLDL were 65.39 ± 7.48 *μ*g/ml, 1.24 ± 0.49 mmol/L, and 0.46 ± 0.10 mmol/L in the HBV group and 41.02 ± 6.88 *μ*g/ml, 0.76 ± 0.21 mmol/L, 0.29 ± 0.05 mmol/L in the normal control group, respectively, with statistically significant differences (*P* < 0.05), as shown in Fig. 4a–c.

## Discussion

HepG2.2.15 cells are HepG2 cells that are stably transfected with the whole HBV genome, which can express all of the RNA and proteins of the virus and secrete virus-like particles [14]. Norton PA et al. screened for genes with differential expression in HepG2.2.15 and HepG2 cells by a DNA microarray and found that the expression levels of ApoA1, ApoB and ApoC3 in HepG2.2.15 cells were decreased [15]. Jiang W and Wang Y confirmed the regulatory effect of HBV on ApoA1 [10, 16]. Our previous study also demonstrated the molecular mechanism of HBV inhibition of ApoB expression and found that HBV can inhibit the expression of ApoA5 [9, 11]. In this study, we found that the expression levels of ApoC3 mRNA and protein in HepG2.2.15 cells were lower than those in HepG2 cells, which is consistent with the microarray results of Norton PA. We also found that the expression levels of the ApoC3 promoter, mRNA and protein were inhibited by the transfection of the HBV-infected clone pHBV1.3 and HBV X gene. The examination of clinical specimens confirmed the regulatory effect of HBV on ApoC3 and showed that the serum ApoC3 level in HBV patients was decreased, indicating that HBV can inhibit the in vitro and in vivo synthesis and secretion of ApoC3.

The liver plays an important role in lipid metabolism, and it is not only the main site of lipid synthesis, transport, metabolism, and degradation but also an important site of lipoprotein degradation. A damaged or inflammatory state of liver cells can certainly influence lipid metabolism [17]. HBV infection will illicit the liver’s inflammatory response by up-regulating the expression of inflammatory cytokines, such as interleukin (IL)-6, IL-12, IL-27 and IL-35, resulting in damage to liver cells, thus affecting the metabolism function of the liver and leading to abnormalities in lipoprotein synthesis [18–20].

Studies had shown that the blood lipid levels of HDL-C, LDL-C and TC in patients with HBV infection are significantly lower than those in healthy subjects [21, 22]. HBV can inhibit the expression of ApoA1 by inhibiting the activity of the ApoA1 promoter and inducing the hypermethylation of the ApoA1 promoter [16]. HBV can down-regulate the expression of ApoB by inhibiting the expression of microsomal triglyceride transfer protein (MTP) [9]. HBV inhibits the expression of ApoA5 through its core gene [11]. In this study, we found that HBV could inhibit the expression of ApoC3 at the promoter, transcription and translation levels via its X gene. Accumulating evidence implied that HBX induces hypermethylation in the promoter region of many genes such as E-Cadherin, insulin-like growth factor-3, caveolin-1 and CD82 which results in their suppression [23–26], thus, HBV may inhibit the expression of ApoC3 through inducing hypermethylation of its promoter via HBX, and the detailed molecular mechanisms needs further investigation.

Subsequently, we compared the serum levels of TG and VLDL in HBV patients and normal controls and found that the serum levels of TG and VLDL were lower in HBV patients. ApoC3 is a lipoprotein lipase (LPL) inhibitor, which can inhibit the activities of lipase, hepatic lipase and lecithin cholesterol acyltransferase to affect lipid metabolism [27, 28]. LPL is a key enzyme in the catabolism of triglyceride lipoprotein (TRL) [29, 30]. When the ApoC3 expression decreases, the activity of LPL increases, the synthesis and secretion of VLDL decreases, and the decomposition of TG increases [31–33]. Therefore, the decreased serum levels of TG and VLDL in HBV patients may be related to the inhibition of ApoC3 expression by HBV.

### Limitations

However, there are two limitations of this study. Firstly, ApoC3 has many genetic polymorphic sites, which can affect the serological levels of TG and VLDL [7, 34]. For example, the T3206G polymorphism of the APOC3 gene is closely related to the blood lipids in hypertriglyceridemia, and the serum level of TG in ApoC3 G3175G genotype carriers is higher than those of other genotypes [35]. This study did not compare the differences in these ApoC3 polymorphisms in the HBV patients and normal controls. Secondly, other confounders related to lipid levels such as the habitual diet of the participants were not available, we could not include such variables in analysis. More rigorous design would be performed in the future study.

## Conclusion

In conclusion, we investigated the regulatory effect of HBV on the expression of ApoC3 at the cell level and clinical level for the first time, laying the foundation for revealing the pathogenesis of HBV.

## Abbreviations

ALT: Alkanine aminotransferase
ApoC3: Apolipoprotein C3
AST: Aspartate aminotransferase
BMI: Body mass index
CD82: Cluster of differentiation82
ECL: Electrochemiluminescence
ELISA: Enzyme-linked immunosorbent assay
HBV: Hepatitis B virus
HDL-C: High-density lipoprotein cholesterol
Interleukin: IL
LDL-C: Low-density lipoprotein cholesterol
LPL: Lipoprotein lipase
n: number of the subjects
NC: Nitrocellulose
PBS: Phosphate-buffered saline
RT-qPCR: Real-time quantitative polymerase chain reaction
SDS-PAGE: Sodium dodecyl sulfate–polyacrylamide gel electrophoresis
TC: Total cholesterol
TG: Triglyceride
TG: Triglyceride
TRL: Triglyceride lipoprotein
VLDL: Very-low-density lipoprotein
